# On the physics of chromatin molecular complex: from phase-separation to membrane adsorption

**DOI:** 10.3389/fmolb.2026.1951961

**Published:** 2026-10-12

**Authors:** Andrea Fontana, Andrea M. Chiariello

**Affiliations:** Dipartimento di Fisica, Università Degli Studi di Napoli Federico II, and INFN Napoli, Complesso Universitario di Monte Sant’Angelo, Naples, Italy

**Keywords:** chromatin 3D architecture, computational biology, membrane adsorption, phase-separation, polymer physics

## Introduction

Genome organization within the cell nucleus is closely linked to essential cellular functions, including gene expression, DNA replication and DNA repair (Dekker and Mirny, 2024; Willemin et al., 2024; Bantele et al., 2025; Dekker et al., 2025). It is therefore crucial to understand the rules that dictate how chromatin three-dimensional (3D) architecture facilitates communication between genes and distal regulatory elements (Liang et al., 2026), such as enhancers, that may be located far apart along the chromatin fibre.

A substantial body of experimental and theoretical work has highlighted the fundamental role of physical processes (Fiorillo et al., 2020), e.g., phase-separation, chromatin polymer loop-extrusion and membrane adsorption, in shaping chromatin structure and its consequent impact on gene regulation and expression. Phase-separation (Fontana et al., 2026a), i.e., that process through which proteins, and other biomolecules, interact with themselves to form stable condensates, is a fundamental mechanism that recurrently occurs within the cell nucleus and contributes to several biological processes. These include, e.g., mediating long-range contacts between genes and regulatory elements (Boija et al., 2018; Sabari et al., 2018), the formation of large condensates, such as Cajal bodies (Banani et al., 2017; Shin and Brangwynne, 2017), or the organization of multi-phase structures, such as nucleoli (Quinodoz et al., 2025), which consist of distinct molecular species. Notably, alteration in the phase-separation properties of proteins can contribute to disease development (Ahn et al., 2021; Alberti and Hyman, 2021; Mensah et al., 2023).

Another relevant mechanism is loop-extrusion, an active process in which molecular motors, typically cohesin or condensin (Davidson et al., 2019; Kim et al., 2019; Banigan and Mirny, 2020), slide along the chromatin fibre and are halted at specific anchor points, often corresponding to CTCF-binding sites (Sanborn et al., 2015; Fudenberg et al., 2016; Goloborodko et al., 2016; Ganji et al., 2018; Vian et al., 2018). This process contributes to global chromosome condensation (Davidson et al., 2023; Sabaté et al., 2025), as well as local chromatin domains and loops formation (Dey et al., 2023; Hafner et al., 2023). Loop-extrusion is also implicated in chromatin re-wiring following genome perturbations, such as structural variants, e.g., deletions or inversions (Chudzik et al., 2025; Salnikov et al., 2025).

On top of that, the nuclear envelope represents another important layer of chromatin 3D organization (Briand and Collas, 2020; Ringel et al., 2022; Czapiewski and Schirmer, 2024). Specifically, through interactions with peripheral chromatin, nuclear lamina protein network, embedded in the membrane, promotes DNA spatial segregation from the inner nuclear environment, with important consequences for gene activity (Chang et al., 2022; Pujadas Liwag et al., 2024).

In this opinion, we discuss recent advances in theoretical and computational models developed to investigate these physical processes within the nucleus of cells, with a particular focus on the interplay between different mechanisms when they are integrated into the same framework, as well as on their relationship with chromatin 3D organization and gene regulation. First, we review recent approaches that quantitatively model phase-separation of molecular aggregates and examine how this process plays a role in the formation of contacts between fundamental classes of genes and their distal regulatory elements (Chiariello et al., 2020; Guha et al., 2025), as well as how it can influence chromatin architecture at the scale of entire chromosomes (Rossi et al., 2025). Next, we consider models that incorporate the nuclear membrane, which are particularly relevant for describing chromatin structure at the nuclear periphery, as shown in recent studies (Ringel et al., 2022; Chudzik et al., 2025). Applications of these models to the understanding of specific genome functions and how their alteration may contribute to disease conditions is finally discussed.

## Models of phase-separation: a fundamental mechanism involving proteins and chromatin

It is well established that the formation of phase-separated condensates can be triggered by the self-interaction of proteins, typically mediated by their intrinsically disordered regions (IDRs) (Shin and Brangwynne, 2017). Important examples of proteins capable of such homo-typic interactions include the Mediator and RNA polymerase II (RNA PolII) (Cho et al., 2016; 2018; Budinich et al., 2026), coactivators (Sabari et al., 2018) and transcription factors (TFs) (Boija et al., 2017; 2018). In addition, condensates can contain multiple molecular species and be stabilized by more complex networks of interactions, as occurs, for example, in the nucleolus through interactions between proteins and RNA (Shin and Brangwynne, 2017; Riback et al., 2020).

Given the increasing number of experimental studies showing that phase-separation plays a crucial role in specifically defining chromatin structures and regulating gene activity, theoretical and computational models describing systems composed of proteins and chromatin, with the latter represented as a polymer chain, have been developed (Fiorillo et al., 2020). Some approaches consider only polymer-proteins interactions, without explicitly including protein-protein interactions (Barbieri et al., 2012; Brackley et al., 2013; Jost et al., 2014; Chiariello et al., 2016). In this case, the formation of phase-separated protein condensates is induced by their interaction with the polymer and the resulting process is commonly referred to as polymer-polymer phase-separation (Erdel and Rippe, 2018; Erdel et al., 2020; Ryu et al., 2021; Arnould et al., 2023). Although very simple, these models have been successfully applied to re-construct 3D structure of real genomic regions and to describe chromatin re-modelling across different cell types (Brackley et al., 2016; Buckle et al., 2018; Brackey et al., 2020; Chiariello et al., 2020; Forte et al., 2023; Tafuri and Chiariello, 2023), as well as following different viral infections (Chiariello et al., 2024; Fontana et al., 2025b; 2025a; 2026b).

To simulate more realistic biological systems, these models can be made more general by incorporating explicit protein-protein interactions as well as loop-extrusion, as illustrated in Figure 1a. In this case, the system physical behavior is governed by protein concentration, protein-protein self-interaction affinity, chromatin-protein affinity and, finally, density and processivity of extruders. Therefore, the equilibrium state of the system depends on the interplay among these different mechanisms, which collectively act to determine the physical properties of the system and give rise, from the physics point of view, to complex phase diagrams.

**FIGURE 1:**
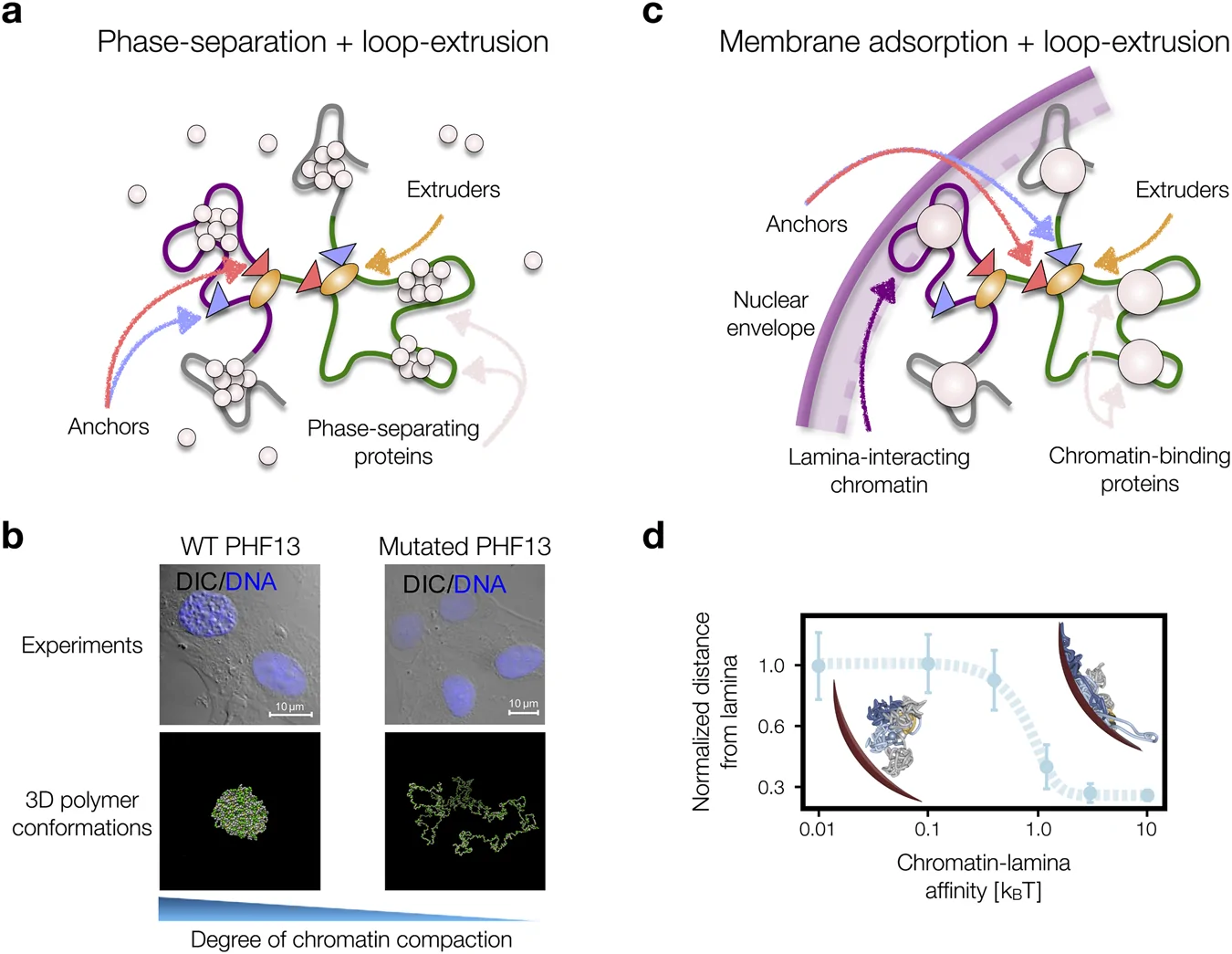
Interplay among different physical mechanisms shaping chromatin 3D organization. **(a)** Computational model combining loop-extrusion mechanism, chromatin-protein interactions and protein-protein phase-separation. Chromatin 3D structures depend on proteins affinities and concentration, as well as on extruders density and processivity. **(b)** Examples of DNA microscopy of interphase cells overexpressing wild-type (WT) self-interaction competent PHF13 (top left) or a self-interaction deficient PHF13 variant (top right). Below, representative 3D polymer conformations obtained from MD simulations are shown. Adapted from (Rossi et al., 2025). **(c)** Computational model explicitly including the nuclear envelope as an adsorbing membrane. Lamina-interacting chromatin regions are attracted towards the nuclear periphery, while chromatin-protein interactions and loop-extrusion simultaneously occur. **(d)** Normalized chromatin distance from the nuclear lamina as a function of chromatin-lamina interaction affinity. At low affinity, chromatin remains predominantly separated from the nuclear envelope (bottom left 3D conformation), whereas increasing attraction drives its progressive recruitment towards the lamina and stabilizes a membrane associated state (top right 3D conformation). Example 3D structures adapted from (Ringel et al., 2022).

Recent studies based on these models have focused, e.g., on the interplay between binding affinities (Ancona and Brackley, 2022), the impact of phase-separation on gene-enhancer communication (Chiariello et al., 2020), the condensation of entire chromosomes driven by protein-protein phase-separation (Rossi et al., 2025), contact re-wiring induced by genetic mutations (Barbieri et al., 2017; Zhang et al., 2023; Platania et al., 2024; Xie et al., 2024) and the relationship between phase-separation and loop-extrusion (Guha et al., 2025; Rashid et al., 2026). To investigate the properties of such complex systems, large-scale molecular dynamics (MD) simulations are typically employed. This represents a powerful numerical strategy, as simulations allow to study both equilibrium states of the system, as a function of relevant control parameters, and the dynamical processes through which these states are reached.

For instance, MD simulations have been employed to investigate the formation of phase-separated protein condensates in the presence of a polymer representing the chromatin filament (Chiariello et al., 2020) and to understand how this latter influences this process. These simulations predicted that, depending on model parameters, phase-separated condensates can either form spontaneously and independently of the polymer or be induced by the polymer itself. This latter scenario occurs when protein-protein affinity is not sufficiently strong to drive phase separation on its own, while the chromatin-protein affinity is sufficiently high (Chiariello et al., 2020), and has subsequently been the subject of recent theoretical (Sommer et al., 2022) and computational studies. Because of its distinctive properties, it has been termed polymer-assisted condensation (PAC) and proposed as a potentially general mechanism (Schiessel, 2022; Mukherjee et al., 2026) relevant to chromatin organization.

A realistic application of a model including both protein-protein and chromatin-protein interactions has been recently employed to investigate PHF13, a protein known to interact frequently with chromatin enriched in the histone methylation marks H3K4me3 and H3K27me3 (Fuchs et al., 2017). In addition, PHF13 can interact with itself through specific sub-domains located in its N-terminal region (Rossi et al., 2025). Simulation of this system showed that, when both chromatin-protein and protein-protein interactions are enabled and the concentration of PHF13 is sufficiently high, the polymer stabilizes into a compact, approximately spherical structure, with PHF13 clusters interacting with the chromatin fibre. Importantly, this result is consistent with microscopy experiments performed in interphase cells overexpressing PHF13, in which chromosomes condense into rounded structures (Rossi et al., 2025). Furthermore, when protein-protein interactions are disabled, polymer compaction is reduced and chromosome condensation is no longer observed, again consistent with microscopy data from cells expressing engineered PHF13 variants lacking the N-terminal self-interacting domains (Rossi et al., 2025), as shown in Figure 1b.

Along the same lines, an analogous model has recently been employed to investigate NUP98-HOXA9 properties, a chimeric protein associated with leukemia (Ahn et al., 2021; 2025; Li et al., 2025). Specifically, NUP98-HOXA9 can undergo phase-separate and bind to chromatin, thereby promoting the formation of aberrant chromatin loops between oncogenes, e.g., *PBX3*, and regulatory elements (Ahn et al., 2021). Modelling of specific genomic regions accurately recapitulates such NUP98-HOXA9 aberrant chromatin looping patterns observed in experimental Hi-C contact data from human cells (Ahn et al., 2021), single molecule mobility measurements (Ahn et al., 2021) and the interplay between phase-separation and loop-extrusion mechanisms (Guha et al., 2025; Rashid et al., 2026).

### Chromatin and nuclear lamina: tuning gene-regulation

Another biological component that is fundamental to chromatin organization and gene regulation is the nuclear scaffold, which provides an additional layer of architectural control for large chromatin regions located at the nuclear periphery (Poleshko et al., 2017; Ringel et al., 2022). The association of chromatin with the nuclear lamina complex is extensively re-modelled during cellular differentiation and following genomic perturbations, with corresponding changes in gene activity. From the physics point of view, an intuitive approach to study these systems is to employ computational models that explicitly represent nuclear lamina as an adsorbing membrane. Specifically, the membrane can interact with chromatin through attractive or steric interactions, which may be assigned on the basis of broad genome segmentations (Falk et al., 2019) or experimental data providing locus-specific information on lamina association. To this end, experimental DamID profiles, which quantify such lamina association, can be used to parameterize chromatin-lamina interactions, e.g., by simply introducing thresholds on the DamID signal: if above threshold, the genomic region attractively interacts with membrane, only sterically otherwise (Ringel et al., 2022).

As discussed above, loop-extrusion and chromatin-protein interactions can also be incorporated into the same modelling framework, as shown in Figure 1c. The resulting model provides an accurate description of loci associated with the nuclear lamina and organized by CTCF-binding sites, as recently demonstrated, for example, for the mouse genomic region containing the *Sox9* and *Kcnj2* genes (Chudzik et al., 2025). Importantly, different degrees of lamina association can be reproduced by tuning the chromatin-lamina affinity (Figure 1d) (Divisato et al., 2022; Ringel et al., 2022), which in turn can be optimized to describe distinct cell populations classified according to the expression levels of the associated genes (Chudzik et al., 2025). Notably, MD simulations have also been used to investigate the effects of genomic editing on chromatin-lamina association and revealed a strong quantitative agreement with experiments involving the deletion of CTCF-boundary elements (Chudzik et al., 2025).

Overall, these findings established a link between loop-extrusion and chromatin-lamina association, which has been successfully captured by the model even though the two physical processes occur independently within the system. Moreover, protein phase-separation can also be incorporated into these models with membrane adsorption, thereby providing a computational framework capable of describing an even greater degree of biological complexity.

## Discussion

In this opinion, we highlighted the versatility of theoretical and computational models based on physical mechanisms occurring in the cell nucleus for investigating chromatin three-dimensional organization and gene regulation. We focused on fundamental physical mechanisms, including phase-separation, membrane adsorption and loop-extrusion, and discussed recent model variants that integrate combinations of these mechanisms within a single computational framework (Chiariello et al., 2020; Chudzik et al., 2025; Guha et al., 2025; Rossi et al., 2025).

The main advantage of these approaches is their ability to describe increasingly detailed biological contexts, although this comes at the cost of larger control parameter spaces and more complex phase diagrams. Such conceptual and computational challenges are well tackled through molecular dynamics simulations, which effectively illustrate how phase-separation, loop-extrusion and membrane adsorption mechanisms can act cooperatively to explain the behaviour of several, different biological systems, often in close agreement with experimental observations.

These models have already provided novel insights into several biological scenarios, including the influence of chromatin on the induction of phase-separation (Chiariello et al., 2020), chromosome condensation triggered by protein phase-separation (Rossi et al., 2025), the interplay between loop-extrusion and protein phase-separation (Guha et al., 2025) and the impact of boundary elements mutations on chromatin-lamina interactions (Chudzik et al., 2025). Further applications of these approaches may extend to other biologically relevant conditions, including pathogenic protein variants, ageing, and alterations in chromatin architecture induced by external pathogens, such as viruses (Fontana et al., 2026b).
